# Medical students' preparation for the transition to postgraduate training through final year elective rotations

**DOI:** 10.3205/zma001142

**Published:** 2017-11-15

**Authors:** W. E. Sjoukje van den Broek, Marjo Wijnen-Meijer, Olle Ten Cate, Marijke van Dijk

**Affiliations:** 1 University Medical Center Utrecht, School of Medicine, Utrecht, The Netherlands; 2 University Medical Center Utrecht, Centre for Research and Development of Education, Utrecht, The Netherlands

**Keywords:** Undergraduate Medical Education, Continuing Medical Education

## Abstract

**Objectives:** This study adds to the ongoing discussion on how to ease the transition from undergraduate medical training to postgraduate training. In the Netherlands there is no central matching system for admission to residency. Medical school graduates just apply for a position in an open job market. Many choose to acquire general or specialty-specific clinical experiences after the medical degree before residency, to further explore career opportunities and to increase their chances to get into their preferred specialty. To shorten this gap between undergraduate and the start of postgraduate training, the sixth and final year of most Dutch medical schools is designed as a “transitional year”. Students work with more clinical responsibilities than in the earlier clerkships, and this year includes many elective options. Our study focuses on these elective options and explores how medical students use these transitional year electives to prepare for transition to postgraduate training.

**Methods:** In 2012-2013 we asked all 274 graduating students at one Dutch medical school to complete an open-answer questionnaire with the following topics:

1. their preferred specialty at the start of the transitional year,
2. electives they chose during this year and reasons for these choices, and
3. whether the transitional year electives changed their career considerations.

Questionnaire results were coded by two researchers and were discussed with all members of the research team.

**Results:** A total of 235 students responded (86%). Answers about motivation for choices revealed that most electives where chosen for career orientation and to optimize chances to get into a residency program. Students also focused on additional experiences in specialties related to their preferred specialty. Many students chose electives logically related to each other, e.g. combinations of surgery and radiology. About two-thirds of the respondents stated that their elective experiences did confirm their specialty preferences or resulted in a more clear insight.

**Conclusion: **We conclude that students use the transitional year electives to focus on their future postgraduate training program, i.e. for orientation and to align their curriculum vitae with their preferred specialty, resulting in spontaneous early specialty streaming. To take advantages of this streaming, and to make sure students can transfer their experiences to other specialties if their career preferences change, individual elective Entrustable Professional Activities (EPAs), next to the core EPAs for all medical students, may serve to prepare a smooth transition to a specialty of choice and should be fully documented.

## Zusammenfassung

**Ziele: **Dies ist ein Beitrag zu der laufenden Diskussion darüber, wie man den Übergang vom medizinischen Grundstudium zu der weiterführenden Ausbildung erleichtern kann. In den Niederlanden gibt es kein zentrales Matching-System für die Zulassung zur Assistenzzeit. Medizinabsolventen bewerben sich einfach auf dem offenen Stellenmarkt um eine Stelle. Viele entscheiden sich dafür, nach dem Medizinexamen vor der Assistenzzeit allgemeine oder fachspezifische klinische Erfahrungen zu sammeln, Karrieremöglichkeiten näher kennenzulernen und ihre Chancen auf ihr gewünschtes Fachgebiet zu erhöhen. Um die Zeit zwischen dem Grundstudium und dem Beginn der Postgraduatiertenausbildung zu verkürzen, ist das sechste und letzte Jahr der meisten holländischen medizinischen Hochschulen als „Übergangsjahr“ konzipiert. Die Studenten arbeiten mit größerer klinischer Verantwortung in den Kliniken und können in diesem Jahr viele Wahlmöglichkeiten nutzen. Unser Beitrag konzentriert sich auf diese Wahlmöglichkeiten und untersucht, wie die Medizinstudenten die Wahlmöglichkeiten dieses Übergangsjahres nutzen, um sich auf die Postgraduiertenausbildung vorzubereiten.

**Methoden:** 2012-2013 baten wir alle Studenten einer holländischen medizinischen Hochschule, einen Fragebogen mit folgenden Themen auszufüllen:

1. das von ihnen gewünschte Fachgebiet zu Beginn des Übergangsjahres,
2. Wahlfächer, die sie während dieses Jahres wählen und ihre Gründe dafür und
3. ob die Wahlfächer in dem Übergangsjahr die Überlegungen zu ihrer Laufbahn verändert haben.

Die Antworten des Fragebogens wurden von zwei Forschern kodiert und unter allen Mitgliedern des Forschungsteams diskutiert.

**Ergebnisse:** Insgesamt haben 235 Studenten (86%) geantwortet. Aus den Antworten bezüglich der Wahlmotivation ging hervor, dass die meisten Wahlfächer aus Karrieregründen gewählt wurden, bzw. um die Chancen auf einen Platz im Assistentenprogramm zu erhöhen. Darüber hinaus waren den Studenten zusätzliche Erfahrungen in Fächern wichtig, die mit ihrem gewünschten Fachgebiet zusammenhängen. Viele Studenten wählen logisch miteinander verbundene Wahlfächer, z.B. die Kombination von Chirurgie und Radiologie. Etwa zwei Drittel der Antwortenden sagten, dass ihre Wahl ihren Wunsch nach einem bestimmten Fachgebiet bestätigt oder ihnen einen klareren Einblick verschafft habe.

**Schluss: **Wir schließen daraus, dass die Studenten die Wahlfächer des Übergangsjahres dazu nutzen, sich auf ihre Postgraduatiertenausbildung zu konzentrieren, d.h. sich zu orientieren und ihr Curriculum an ihr gewünschtes Fachgebiet anzupassen, woraus sich eine spontane frühe Spezialisierung ergibt. Um diese Tendenz zu nutzen und sicherzustellen, dass die Studenten ihre Erfahrungen auf andere Fächer übertragen können, wenn sich ihre Laufbahnwünsche ändern, können individuelle Entrustable Professional Activities (EPAs), die man neben den für alle Medizinstudenten vorgeschriebenen Kern-EPAs wählen kann, zu einem reibungslosen Übergang zu dem gewünschten Fachgebiet verhelfen. Sie müssen genau dokumentiert werden.

## 1. Introduction

The continuum of undergraduate and postgraduate medical education is becoming more important, while the importance of the medical degree decreases [1]. Therefore, it is increasingly relevant to understand the dynamics of orientation and preparation for residency during medical school. The final year of medical school could be of major importance for this purpose. The structure and content of this year, and how this year could successfully prepare students to enter postgraduate education, is therefore a topic of debate [2], [3]. 

Differently than in for instance the USA, the Netherlands has no national matching system for residency training programs. Medical graduates just apply for a position in an open job market. Many graduates choose to acquire general or specialty-specific clinical experiences after the medical degree and before applying for residency training. Or they try to obtain a PhD position, to further enhance career opportunities, and to increase the chances to get into their preferred specialty training program. A survey in 2013 revealed that there is a time lag of 28,5 months on average between the medical degree and starting residency in the Netherlands, outliers excluded [4]. There is widely held opinion that this intervening period needs to be shortened, to decrease the total training time from secondary school student to medical specialist, and to allow specialists to practice for a longer period of their lives. One way to reduce the length of the intervening period is to better gear medical school towards postgraduate training. The final year of most medical schools in the Netherlands is designed as a so-called “transitional year” since about one decade [5], [6]. During this year, which is part of undergraduate training, students work with more clinical responsibilities than in the earlier clerkships, at the level of a starting resident under strict supervision. This increase in clinical responsibilities for students has shown to improve graduates preparation for residency at the level of their skills and knowledge. In a questionnaire study among supervisors of postgraduate training programs, graduates from a curriculum with such a structure were judged to be more capable to work independently, to solve clinical problems, to manage unfamiliar medical situations, to prioritize tasks, to collaborate with other professionals, to judge when they need help from their supervisors and to reflect on their activities [7]. 

In addition, also in order to stimulate the transition into residency, the transitional year includes many elective options, with the intention to have students choose specialties they want to gain more in-depth experience in. While students can gain more knowledge and skills in their areas of interest, supervisors may observe and identify future applicants for specialty training [6], [8]. Recently, the Dutch government has demanded to shorten the medical training continuum within the constraints of the EU rules. One intervention that is being introduced is to change the “transitional year” with its electives into a “dedicated transitional year”. This means that students focus on a specific future residency training program, by spending their final year at a set of predetermined rotations, in most cases limited to a specific specialty. After successful completion these junior doctors can participate in a program of reduced length for the first year of residency. It is not clear yet how useful this approach will be. It is common sense among students that the success of the transitional year with electives is in the creation of individual pathways. 

In the light of these developments on the structure of the final year of undergraduate training, we aimed to explore whether and which spontaneous specialty streaming emerges in elective choices of medical students during their final year, what students’ rationales are in choosing these electives, and how considerations for residency training change during the final year electives, in the context of a transitional year program. 

## 2. Methods

### 2.1. Context: The Transitional Year program at Utrecht Medical School

Our study was conducted at one Dutch medical school with a transitional year program, namely Utrecht University medical school. The transitional year at this medical school was introduced in 2004. Students start the transitional year with a mandatory 6-week training course in evidence based medicine, medical professionalism and teacher training. Then, in a varying order, students must take a 12-week major clinical elective at a department of their choice, a 12-week research elective at a department of their choice, and have 12-weeks to fill in freely. Students can take these 12-weeks additional electives at any clinical, science or other department they want, or choose for a teaching rotation, health management or medical ethics elective. Splitting up in two times six weeks is also allowed for these 12-week additional electives. For the 12-week major clinical elective, choices are restricted to make sure students will obtain experience with a sufficiently broad range of clinical problems. Departments students can choose from include family medicine, general internal medicine, general surgery, geriatrics, neurology, obstetrics and gynecology and pediatrics. Ophthalmology, ENT and anesthesiology are among the specialties that cannot serve as a major clinical elective, but are allowed as departments for an additional research or clinical elective in the 12 weeks students can fill in freely.

#### 2.2. Population and instrument

We asked all 274 students who graduated between July 2012 and July 2013 from Utrecht University medical school to participate. All students participated voluntary and signed an informed consent form. Ethical approval for this study was obtained from the ethical review board of the Netherlands Association for Medical Education. Around graduation, all participants were asked to fill out an open-answer paper-based questionnaire about their specialty preferences at the start of the transitional year, their elective choices with motivation during this year, and whether their specialty preferences had changed and how. We choose a paper-based questionnaire to stimulate response rate. We choose open-ended questions, in order to explore the full width of considerations students have about their elective choices. 

#### 2.3. Data analysis

Each specific area of specialty where students did electives was given a code, as well as for the electives in other fields such as the teaching rotation or medical ethics. We grouped some specialties, such as surgical subspecialties to “surgery” and internal medicine subspecialties to “internal medicine”, in discussion with all members of the research team. We used Dedoose® version 6.1.18 to support our data analysis and to generate an overview of the co-occurrence of electives in elective tracks of respondents to the questionnaire.

Answers about motivation for choosing an elective, and whether and how specialty preference had changed during the transitional year, could be interpreted more subjectively and coding of these answers therefore involved five consecutive steps. The first step involved the review of and familiarization with the data by three researchers (MWM, MD and SB). Stage 2 involved identifying themes by open coding to develop a code framework discussed by MWM, MD and SB. Stage 3 involved a try-out coding for a part of the data by two researchers (MWM and SB), after which the codes were slightly revised. Stage 4 included coding of another part of the data by the same two researchers, after which a satisfactory interrater reliability was reached. Interrater reliability was computed using SPSS Statistics version 20.0 Cohens’ Kappa test for interrater agreement. Stage 5 was the final coding of the data by one researcher (SB), in case of doubt answers were discussed with a second researcher (MWM) and if necessary a third researcher (MD). While coding the data the process was regularly reviewed and discussed with all research team members. 

## 3. Results

A total of 236 students filled out the questionnaire (response rate 86%). One participant was excluded, as this questionnaire yielded unusable data. For coding of the answers about motivation for choosing an elective, a Kappa measure of agreement of 0.82 was reached, and for the answers whether specialty preference had changed during the transitional year a Kappa of 0.69 was found. 

### 3.1. Career interests at start of the transitional year

When asked about specialty preferences, students reported up to six specialties of interest, with a mean of 1.99 (SD 0.99). Of all participants, 153 students reported a specific ranking in their preferences, or reported only one specialty of preference at the start of the transitional year. Internal medicine with its subspecialties was most popular (N=33, 21.6%), followed by family medicine (N=30, 19.6%), surgery (N=24, 15.7%), pediatrics (N=17, 11.1%), and other specialties with percentages below 10. The other 81 participants may have had a ranking in mind while starting their transitional year, but this ranking was not clearly identifiable in their answers. 

#### 3.2. Rationales for choice of transitional year electives

The analysis about rationales for choice of transitional year electives yielded three predominant groups:

1. To orient toward a residency, i.e. try out if, or affirm that a specialty would be a suitable option.
2. To maximize chances to be selected for a postgraduate program of choice, i.e. to either build experience in that specialty, or to obtain experience in related domains presumably highly valued by the program of choice (e.g. neonatology for a gynecology choice). Some students also described a combination of orientation and maximizing their changes. We also included these answers in this category.
3. “other reasons” not related to specific considerations about a future career, for example because it took little effort to arrange a certain elective, or because the students wanted to fill gaps in their knowledge and skills in general.

The major clinical and research electives in the transitional year were mostly used for orientation and maximizing changes to be selected for a postgraduate program (80% and 75% respectively), while for the additional electives other reasons were mentioned more frequently (around 40%). 

#### 3.3. Frequent combinations of electives during the transitional year

Table 1 (Tab. 1) gives an overview of co-occurrence of electives in elective tracks of our respondents. We found some electives to be frequently chosen in combination with specific other electives. For example, 19.4 percent of students who did at least one elective at anesthesiology, also choose at least one elective at the ICU (upper row). 

In addition, 58.3% (N=137) did two, 3.8% (N=9) did three, and 0.4% (N=1) of the students did four electives at the same specialty out of a maximum of 4 different elective periods. 

#### 3.4. Change in considerations for residency during the transitional year

Three groups of considerations to change residency preference after the final year electives were identified:

1. Two-thirds of the students reported that the transitional year confirmed an earlier preference for a residency, or resulted in a more clear insight into their preferences.
2. One sixth of the students reported to still be in doubt when finishing the transitional year.
3. One sixth of the students reported that the elective experiences had caused substantial doubts about their specialty preference or resulted in new insights about their preferences.

## 4. Discussion

The transitional year program was introduced in Dutch medical schools to optimally prepare students for postgraduate training. Next to the increase in clinical responsibilities, an important aspect of the transitional year is that students have a lot of elective choices. We conclude that students use these electives with a strong focus to prepare for residency. These results are not surprising, and resonate with findings of other studies on students’ perspectives on their final year of medical school. Externally driven goals, like identifying a preferred specialty and obtaining a job after graduation, play a major role in students’ choices for the final year [9], [10]. Additionally, we found that when students have a free choice, they choose electives with these purposes, while at the same time making sure that they still gain a broad experience in other specialties. 

Many students still have multiple career interests at the start of the final year. They appear to compose the final year in such a way that they are able to further explore career interests, and at the same time maximize their changes to be selected for a postgraduate training program. In the Netherlands, medical graduates have to apply for residency in an open job market. The shortage of available places for some residency programs may serve as a motivation for elective choices in the transitional year. For example, we found many students choosing a clinical elective in surgery or internal medicine because of its breadth. In the answers on the questionnaires students stated that they see an elective at these departments as an adequate preparation for almost every career option. We also see students choosing electives at departments logically related to each other, such as surgery with radiology, cardiology with Intensive Care, anesthesiology with surgery and Intensive Care, and family medicine with emergency medicine (see table 1 (Tab. 1)). Students mentioned that they see their final year electives as an opportunity to gain additional experiences with related specialties of their specialty of preference, which might not be part of their specialty training, but could be useful in their future career. 

An intervention being introduced in the Netherlands is to reconstruct the final year of medical school to a so-called “dedicated transitional year”. Students must then choose all, or most, final year rotations in a specific specialty direction. After successful completion these junior doctors may participate in a reduced program for the first year of residency of this specialty. The actual effective success of such mandatory early specialty streaming for the training of physicians is not clear. On one hand, students simply get an opportunity to learn in depth in a field they might start working in after graduation, which probably eases their transition to residency. On the other hand, a possible threat is that this could limit the students’ broad development as physician. 

We found that when students have a free choice, they choose electives to prepare for residency while at the same time guaranteeing a broad experience in other specialties. One way to further support this finding is to give students the opportunity to record their individual capacities, apart from the specialty where they did their electives. The registration of their development by for example using Entrustable Professional Activities (EPAs) [11] could be useful for this purpose. Next to the core EPAs for all medical students, individual elective EPAs could be implemented, by which students can develop and show unique profiles [12]. These elective EPAs might be more specialty specific. However, students can work on these EPAs at several departments, and are not necessarily limited to electives at specific specialties. For example, an EPA obtained at a surgical department could be very useful when interested in a future career in dermatology. Students can still strengthen their resumes by working on specific skill development, but they can do this at any department. For most students in this study their elective experiences confirmed their decision for a specialty, or resulted in a more clear insight into their preferences for residency training programs. However, not all of these students can get into the residency they had in mind, given the limited training capacity for some specialties, and also a not insignificant number of students changed their specialty preference. A registration of EPAs could be useful when applying for a job after graduation, also when applying for a specialty that does not match the focus of their electives during the final year. 

Secondly, it is not clear how mandatory early specialty streaming, already at the level of medical school, affects students’ professional development. During medical training, students go through a process of socialization, by which they learn to function within the medical profession by internalizing its values and norms [13]. This process of socialization and developing a professional identity is strongly influenced by external factors, such as role models, and the interaction with patients and peers [14], [15]. It is therefore important to gain insight into the professional identity development of undergraduate medical students, and how this could be affected by mandatory early specialty streaming.

Transitional year programs in Dutch medical schools are part of the regular six years of training time for undergraduate medical education in the Netherlands. Their purpose is educational. Students are not meant to be scheduled in as employees during this year, and do not receive payment. Thus, the transitional year does not create a ‘cheap labor’ environment for junior doctors who have not yet secured a residency position. After successfull completion, students graduate and enter an open job market for residency. However, transitional year programs do give students the opportunity to identify careers early and develop unique profiles, which can serve as stepping stones in their career as young doctors. There are indications that adequate transitional years lead to more rapid admission into residency [16], and that consequently the desire to be employed as a junior doctor in an interim period before residency may decrease.

Our study has strengths and limitations. The open nature of the questions in the questionnaire, in combination with a high response rate, gives a good insight into the considerations of final year students in choosing their electives. However, we conducted this study only for one cohort of medical students at one medical school. Therefore conclusions should be generalized with caution. Students were asked about preferences for residency training at the start of the transitional year, and their reasons for choosing their electives, but we only asked this in retrospect. Students might have changed their mind about career preferences during the year. This could have resulted in students mentioning other reasons for choosing an elective than actually were the case at the time of making those decisions, or in students switching their elective choices. Also, we do not have information about other activities of students to focus on a future residency training program, such as extracurricular research activities. It is important to realize that the shortage of places for some residency training programs may serve as an extrinsic motivation for elective choices, thus we do not consider elective choices in the transitional year as purely intrinsic considerations. 

This study was meant to be explorative, and to provide insight in medical students’ use of electives during a transitional year to prepare for postgraduate education. To obtain deeper insight whether this indeed results in a better preparation we recommend to follow up cohorts of students. 

## 5. Conclusion

Final year medical students use electives to create experiences that ease the transition from undergraduate to postgraduate medical training programs, resulting in spontaneous early specialty streaming, while still guaranteeing their broad development as a physician. It is not clear whether mandatory early specialty streaming, as is being discussed and starting to be implemented in the Netherlands, will show an improvement on this preparation for residency. 

## Ethical approval

Ethical approval was obtained from the ethical review board of the Netherlands Association for Medical Education, reference number 181, on June 16 2012. 

## Competing interests

The authors declare that they have no competing interests. 

## Figures and Tables

**Table 1:**
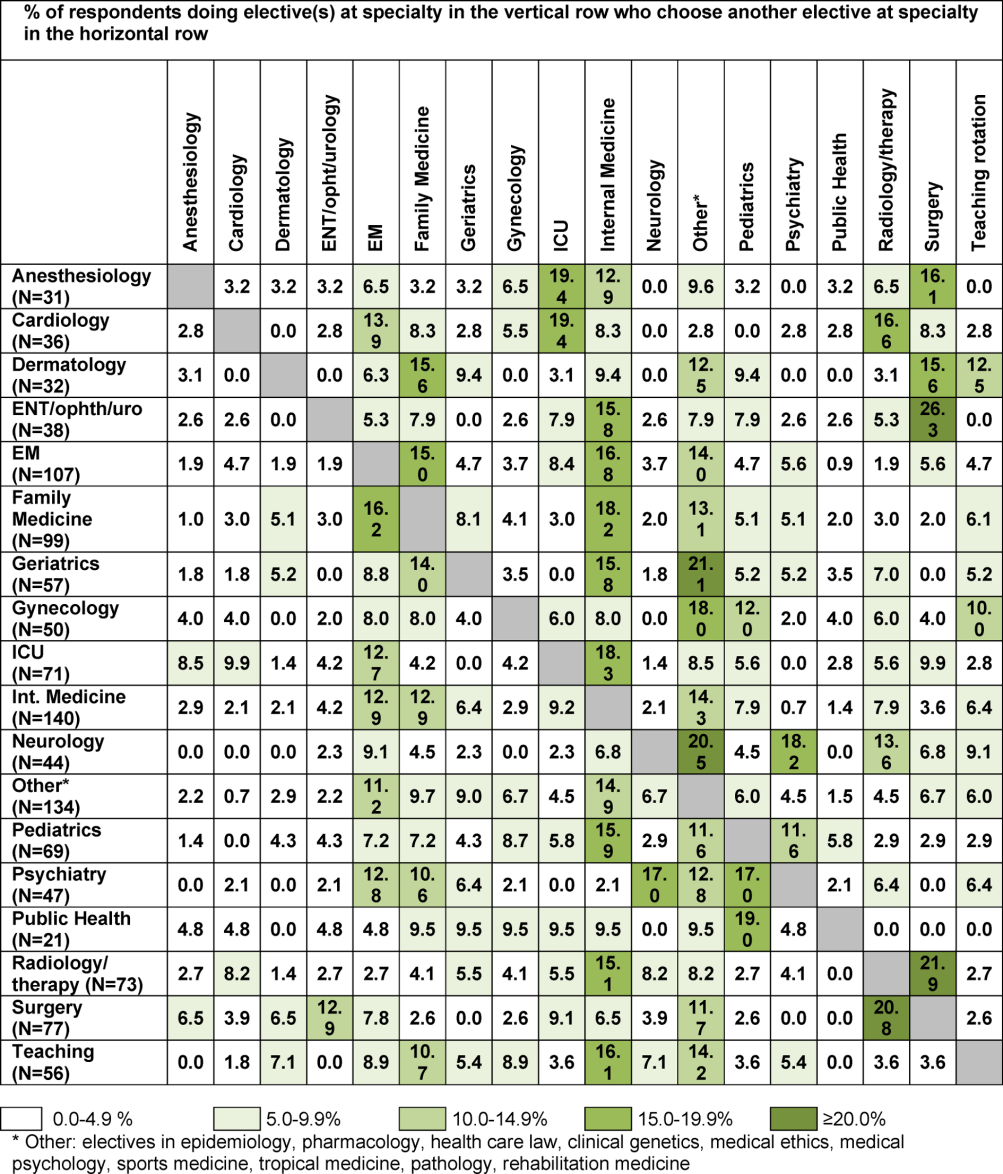
Co-occurrence of electives in elective tracks of respondents
